# Enhancement of cerebrospinal fluid tracer movement by the application of pulsed transcranial focused ultrasound

**DOI:** 10.1038/s41598-022-17314-9

**Published:** 2022-07-28

**Authors:** Seung-Schik Yoo, Hyun-Chul Kim, Jaeho Kim, Evgenii Kim, Kavin Kowsari, Jared Van Reet, Kyungho Yoon

**Affiliations:** 1 Department of Radiology, Brigham and Women’s Hospital, Harvard Medical School, 75 Francis Street, Boston, MA 02115 USA; 2 Department of Artificial Intelligence, Kyungpook National University, Daegu, Republic of Korea; 3 Department of Neurology, Dongtan Sacred Heart Hospital, Hallym University College of Medicine, Hwaseong-si, Gyeonggi-do, Republic of Korea; 4 Department of Mechanical Engineering, Massachusetts Institute of Technology, Boston, MA USA; 5 School of Mathematics and Computing (Computational Science and Engineering), Yonsei University, Seoul, Republic of Korea

**Keywords:** Biotechnology, Neuroscience

## Abstract

Efficient transport of solutes in the cerebrospinal fluid (CSF) plays a critical role in their clearance from the brain. Convective bulk flow of solutes in the CSF in the perivascular space (PVS) is considered one of the important mechanisms behind solute movement in the brain, before their ultimate drainage to the systemic lymphatic system. Acoustic pressure waves can impose radiation force on a medium in its path, inducing localized and directional fluidic flow, known as acoustic streaming. We transcranially applied low-intensity focused ultrasound (FUS) to rats that received an intracisternal injection of fluorescent CSF tracers (dextran and ovalbumin, having two different molecular weights–M_w_). The sonication pulsing parameter was determined on the set that propelled the aqueous solution of toluidine blue O dye into a porous media (melamine foam) at the highest level of infiltration. Fluorescence imaging of the brain showed that application of FUS increased the uptake of ovalbumin at the sonicated plane, particularly around the ventricles, whereas the uptake of high-M_w_ dextran was unaffected. Numerical simulation showed that the effects of sonication were non-thermal. Sonication did not alter the animals’ behavior or disrupt the blood-brain barrier (BBB) while yielding normal brain histology. The results suggest that FUS may serve as a new non-invasive means to promote interstitial CSF solute transport in a region-specific manner without disrupting the BBB, providing potential for enhanced clearance of waste products from the brain.

## Introduction

The brain bears high sensitivity/vulnerability toward changes in the extracellular environment, and demands efficient waste clearance due to its higher metabolic rate compared to the other organs^1,2^. Aberrant waste transport and clearance from the brain has been implicated with aging^3,4^, traumatic brain injury^5,6^, stroke^7–9^, idiopathic normal pressure hydrocephalus^10,11^, and various neurodegenerative conditions, especially with an emerging link to dementia^12^ and Alzheimer’s disease (AD)^13^. Although the exact mechanism of waste transport from the brain is not clearly understood, convective bulk flow, diffusive, or aquaporin-4 channel-mediated (known as ‘glymphatic’) transports, and their combinations mediated by the extracellular fluid in the brain have been identified to play important roles^1–3,14,15^.

The extracellular fluid in the brain consists of cerebrospinal fluid (CSF) and interstitial fluid (ISF) filling the interstitial space^2,15^. The CSF and ISF, along with metabolites and waste products from the brain, are believed to be continuously exchanged/transported through various routes before entering the lymphatic system^1–3,14,15^. The CSF occupies the sub-arachnoid space (SAS) between the arachnoid mater and pia mater, and the pial arteries/veins supply the blood to the brain parenchyma through penetrating vasculature. The blood–brain barrier (BBB), mainly formed by the tight junctions of cerebral vascular endothelial cells and the glia limitans that surround the brain parenchyma, separates the blood circulating in the vasculature from the brain parenchyma while allowing for passive diffusion of water and lipid/water-soluble molecules as well as for active transport of key nutrients^16,17^.

The CSF, separated from blood flow through the blood-cerebrospinal fluid barrier (BCSFB), moves through the perivascular space (PVS) around cerebral arterioles, venules and capillaries, and partially mediates crucial bulk movement of solutes across the brain. Arterial cardiac pulsation was identified as one of the important driving forces behind CSF flow through the PVS^18^, while the numerous microscopic fenestrations on the surface of the pia and leptomeningeal vessels (called ‘stomata’, ~ 3 μm in diameter in humans)^19^ provide potential conduits for CSF and macromolecules/macrophages to reach the PVS^19,20^. The large pial surface and widespread existence of solute transport routes on the cerebral meninges, combined with the mechanical pressure gradient created by arterial pulsation, allow non-diffusional, convective bulk flow of CSF to reach the PVS, which is considered one of the major mechanisms for solute transport and clearance in the brain^18,21,22^.

Insonification of acoustic pressure waves can impose radiation force on a medium in its path, thus inducing localized directional fluidic flow, known as acoustic streaming^23–25^. The acoustic streaming effect has been shown to create convective bulk flow that moves dye molecules through aqueous media in porous, soft tissue-mimicking materials^26^, and is used in applications such as propelling/mixing fluids in microfluidic devices^27–29^, or to detect the presence of fluids in the breast or ovarian cysts^30,31^. Advances in focused ultrasound (FUS) techniques have allowed for non-invasive and targeted transcranial delivery of acoustic pressure waves to a small region of the brain, with flexibility in controlling the depth/location of the acoustic focus^32–37^. Non-thermal mechanical energy conferred by the ultrasound has already been used in various applications such as brain stimulation^38–40^ and drug delivery^36,41^.

We were motivated to examine if acoustic streaming created by the application of transcranial FUS (tFUS) can regionally enhance convective flow of CSF, and thus promotes CSF solute movement in a non-thermal and non-destructive fashion. We first applied FUS in both pulsed and continuous modes to induce fluidic flow of soluble dyes (toluidine blue O, TbO, 270-Da molecular weight–M_w_) in porous media (*i.e*., melamine foam). The pulsing parameter that resulted in the highest dye infiltration was subsequently used in rodent in vivo experiments whereby we applied tFUS to a localized brain area and examined the spatial distribution of intracisternally injected fluorescent CSF tracers (dextran and ovalbumin, having two different M_w_). We hypothesized that regional administration of acoustic pressure waves to the rat brain would facilitate CSF bulk flow into the brain parenchyma, accompanied by the movement of intracisternally injected CSF tracers.

## Results

### FUS transducer and sonication parameters

**Figure 1:**
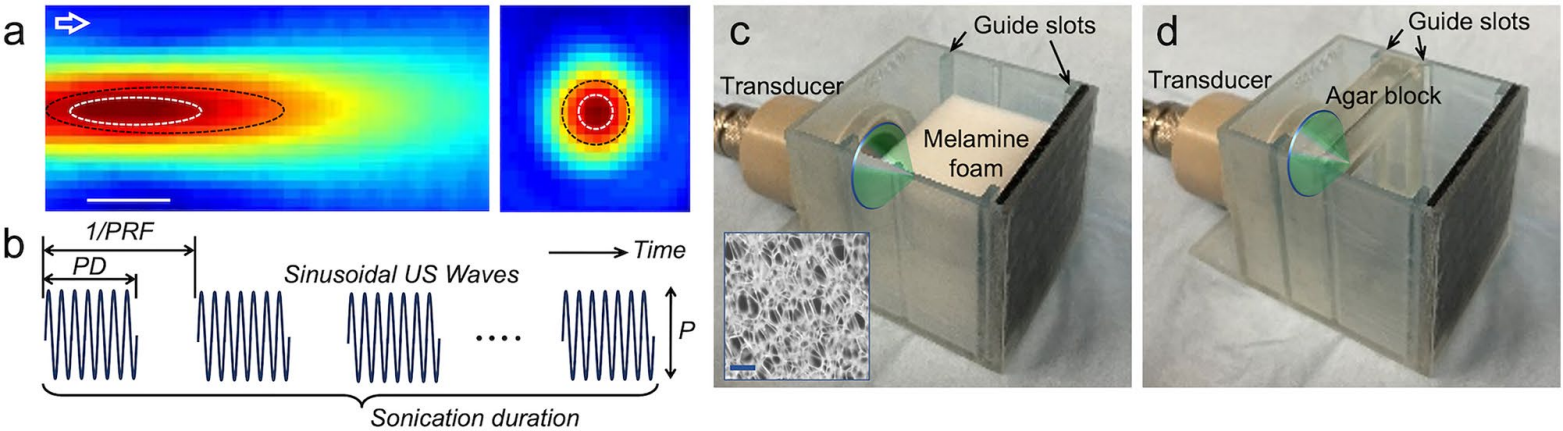
Schematics of the experimental setup and dye infiltration test. (**a**) Pressure profile along the longitudinal and transverse plane of the FUS focus (bar = 10 mm). (**b**) Illustration of the temporal features of sonication parameters used in the experiment. (**c**) Experimental setup for TbO dye infiltration into the melamine block (shown without the TbO solution, inset: microscopic view of the foam, bar = 100 μm). (**d**) A setup for sonicating the agar block shown without the dye solution. The chamber contained guide slots to position the acoustic focus at 1 mm in front of the surface of the block. The green cones illustrate the FUS sonication to the target.

A FUS transducer, operating at a 200 kHz fundamental frequency (FF), generated an acoustic focus 11 mm away from the exit plane of the transducer. The acoustic focus, 5 mm in diameter and a 15 mm long, was defined by the profile bound at full-width at 90% maximum pressure (FW_90%_M; Fig. 1a in white dotted profile), or a 7 mm diameter and a 27 mm length defined at full-width-at-half-maximum (FWHM; black dotted profile in Fig. 1a). The 200 kHz FF was selected considering future transcranial delivery of ultrasound in humans. The schematics and nomenclature of the FUS pulsing parameters are described in Fig. 1b. Bursts of sinusoidal ultrasound waves with defined peak-to-peak pressure amplitudes (P), each of a specific pulse-duration (PD), were administered in a repeated fashion at a pulse repetition frequency (PRF). The PD and PRF together determine the resulting sonication duty cycle (DC) (in %, indicating the fraction of active sonication time per second). The overall duration of pulsed sonication is termed as sonication duration. Acoustic intensity, *i.e.*, acoustic power per given area (W/cm^2^), is expressed in spatial-peak pulse-average intensity (I_SPPA_) while the spatial-peak temporal-average intensity (I_SPTA_) is used to represent its time-averaged value.

### Dye infiltration test

We first examined the feasibility of using acoustic streaming to facilitate fluidic bulk flow into porous melamine foams (pore diameter range of ~ 10–100 μm), which roughly approximated the porosity of cerebral perivascular stomata^19,20^. TbO dye (T3260, Millipore Sigma, Burlington, MA) was dissolved at 25 µg/mL concentration in phosphate buffered saline (PBS, Thermo Fisher, Waltham, MA) for visualization. The foam was hydrated in clear PBS and transferred to a chamber with rubber pad backing (for sound absorption) that contained the dye solution (Fig. 1c, shown without the dye solution).

**Figure 2:**
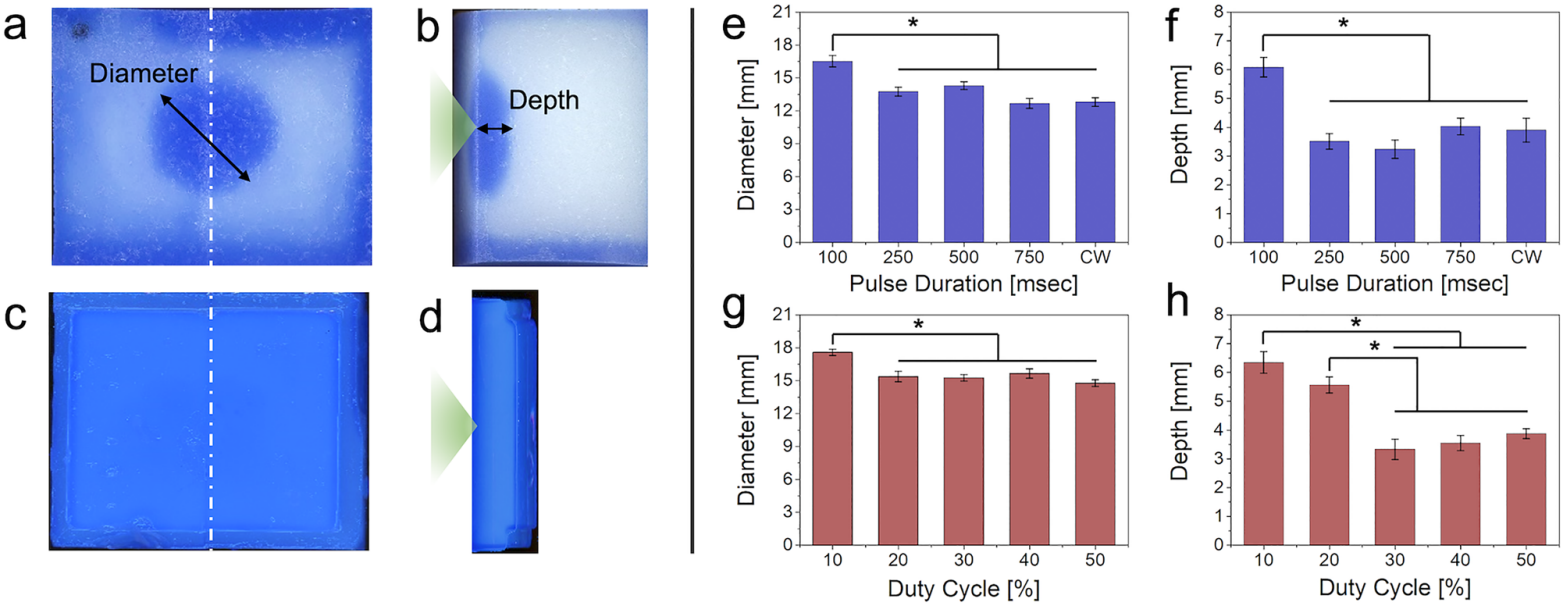
Results from the dye infiltration test. (**a**) Exemplary front view of a melamine foam showing dye infiltration and (**b**) the middle section (dashed line from the front view) showing the dye infiltration depth. The green triangle depicts the direction of sonication. (**c**) Front view and (**d**) the section view of an exemplar agar block that does not show sonication-related dye infiltration. The effects of different pulse durations (PDs in ms; CW = continuous wave) on (**e**) infiltration inlet diameter and (**f**) depth. The effect of FUS given at 100 ms PD with a DC of 10% on the (**g**) inlet diameter and (**h**) depth of TbO infiltration. I_SPTA_ was maintained at 0.5 W/cm^2^ throughout the conditions. Error bar: standard error. *post-hoc Tukey HSD, *P* < 0.001 following ANOVA.

Two sets of experiments were taken in sequence. First, FUS was applied every second (*i.e.,* PRF 1 Hz) to the front of the foam block, with a 1 mm gap between the acoustic focus and the surface of the block, for 10 min using different PDs (100, 250, 500, 750 ms) including a continuous wave (CW) condition. I_SPPA_ was adjusted to 5, 2, 1, 0.67, and 0.5 W/cm^2^, respectively, to maintain an I_SPTA_ of 0.5 W/cm^2^. The corresponding DCs were 10, 25, 50, 75, and 100%. Higher I_SPPA_ values and longer pulse/sonication durations would generate higher acoustic streaming effects^42^, but accompanies a higher susceptibility for heat generation, thus I_SPTA_ was fixed at 0.5 W/cm^2^. The solution was maintained at room temperature (24.2–24.6 °C) during sonication. Following sonication, foams blocks (*n* = 10 in each group) were cut through the midpoint along the sonication direction, then imaged using a flatbed photo scanner. The images were segmented by a threshold intensity of 150 to delineate the area of dye infiltration. Sonication administered at an I_SPTA_ of 0.5 W/cm^2^ yielded visible infiltration on the foam block (example shown in Fig. 2a,b). Image analysis revealed that the use of 100 ms PD, given at a 10% DC at an I_SPPA_ of 5 W/cm^2^ yielded the greatest infiltration diameter over the foam surface (16.5 ± 1.6 mm, average ± standard deviation, *n* = 10) and depth (6.1 ± 1.0 mm) among different PD conditions, including continuous wave (Fig. 2e,f, post-hoc Tukey HSD, P < 0.001 following ANOVA; F(4,45) = 13.3 from diameter measurement and F(4,45) = 11.6 from depth measurement).

Another dye infiltration experiment was conducted using a fixed PD of 100 ms (which generated the highest infiltration in the first set) with different DCs (10, 20, 30, 40, and 50%) by adjusting the PRF to 1, 2, 3, 4, and 5 Hz respectively. To maintain the I_SPTA_ at 0.5 W/cm^2^ across different DCs, I_SPPA_ was adjusted to 5, 2.5, 1.67, 1.25, and 1 W/cm^2^, respectively. We found that the use of a 10% DC given at I_SPPA_ of 5 W/cm^2^ generated the greatest diameter (17.6 ± 0.9 mm) on the foam surface (Fig. 2g with post-hoc Tukey HSD, P < 0.001 following ANOVA; F(4,45) = 20.5). In terms of infiltration depth, both 10% and 20% DCs (given at 5 and 2.5 W/cm^2^ I_SPPA_, respectively) resulted in a greater level of infiltration compared to those from using other DCs (Fig. 2h; post-hoc Tukey HSD, P < 0.001 following ANOVA: F(4,45) = 20.5). The use of a 10% DC yielded the deepest infiltration into the melamine block (6.4 ± 1.1 mm).

We also examined if FUS can facilitate solute movement in a material much denser than melamine foam. The agar hydrogel blocks (*n* = 10), having similar porosity as that of brain parenchyma (40–80 nm)^43,44^, were inserted to a cassette and immersed in the TbO-PBS solution (Fig. 1d shown without the solution). Then, FUS was delivered to the front face of the agar hydrogel block using the sonication parameter that resulted in the highest infiltration in the melamine foam (*i.e.*, 100-ms PD at a 10% DC). The sonication did not have an impact on dye infiltration (0.57 ± 0.10 mm) compared to the unsonicated area (0.54 ± 0.11 mm; paired *t*-test, P > 0.1). The diameter could not be measured in the absence of visible dye marking on the agar surface (Fig. 2c,d).

### Enhanced CSF tracer movement by tFUS

Rats (*n* = 6 in each group, total 3 groups) were anesthetized, then placed on a stereotactic frame to deliver CSF tracers intracisternally based on an established surgical protocol^45^. The CSF tracers were medium M_W_ (45 kDa; similar M_W_ to the neuroactive Aβ oligomers in AD^46^, ~ 8–70 kDa) Texas Red Ovalbumin (OA) and high M_W_ (2000 kDa) fluorescein isothiocyanate (FITC)-dextran (FITC-d), being constituted at a 0.5 wt% concentration in artificial CSF (aCSF).

**Figure 3:**
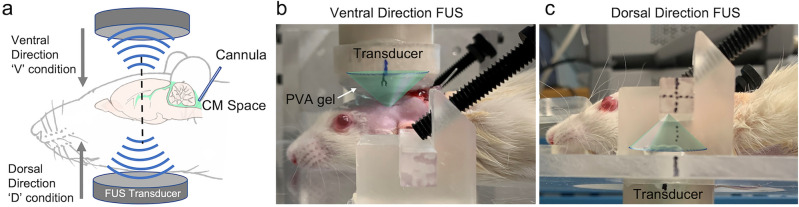
Schematics of the sonication setup for enhancing CSF solute movement in rat brain. (**a**) Illustration of the tFUS setup and the site of the CSF tracer injection. The transducer was mounted to a 2-axis mechanical stage and the animal was positioned over the stereotactic frame that moves vertically. (**b**) Sonication was delivered in the ventral direction through coupling PVA hydrogel and (**c**) to the dorsal direction below the neck. The green cones depict the acoustic profile.

Upon administering the CSF tracers, the animal was positioned on a mechanical sonication setup that stereotactically delivered FUS sonication. With 7 min time gap between end of CSF tracer injection, FUS was transcranially delivered 2 mm caudal to the bregma in ~ 1 mm depth from the top of the head (ventral direction to the brain, noted as ‘V’ condition) as well as from the neck area (dorsal direction to the brain, ‘D’ condition, Fig. 3). A polyvinyl alcohol (PVA) hydrogel was used as a compressible acoustic coupler, and ultrasound gel was applied to all interfaces. When applying FUS through the neck, the transducer was positioned in a manner that avoided the mandibular osseous structures in its path. The sonication was given at a 100 ms PD every second (10% DC) at an I_SPTA_ of 0.5 W/cm^2^ (I_SPPA_ = 5 W/cm^2^; peak rarefactional pressure (P_r_) = 386 kPa) for 30 min. We performed the same experiment without sonication (*i.e.*, the control condition, noted as ‘C’ condition) while maintaining the same time sequence as the sonication experiment.

The animals’ weights were indifferent across the groups (C:V:D = 282.3 ± 17. 0 g: 278.5 ± 14.3 g: 281.7 ± 10.1 g; ANOVA; F(2,15) = 0.126; P = 0.88). The starting heart/respiratory rates were not different across the groups (C:V:D = 218.2 ± 16.4: 217.7 ± 19.8: 220.7 ± 17.8 beats-per-min [bpm], ANOVA; F(2,15) = 0.048; P = 0.95 for the heart rate; C:V:D = 60.7 ± 3.0: 61.3 ± 3.3: 58.7 ± 6.5/min; ANOVA; F(2,15) = 0.556; P = 0.59 for the respiratory rate). The ending heart/respiratory rates were not different across the groups (C:V:D = 216.2 ± 18.4: 217.0 ± 22.7: 217.3 ± 19.7 bpm; ANOVA; F(2,15) = 0.005; P = 0.99 for the heart rate; C:V:D = 59.3 ± 3.0: 57.3 ± 7.3: 58.3 ± 5.3/min; ANOVA; F(2,15) = 0.205; P = 0.82 for the respiratory rate). Heart/respiratory rates did not change before and after the sonication within each condition (paired *t*-test, two-tail, all P > 0.2).

**Figure 4:**
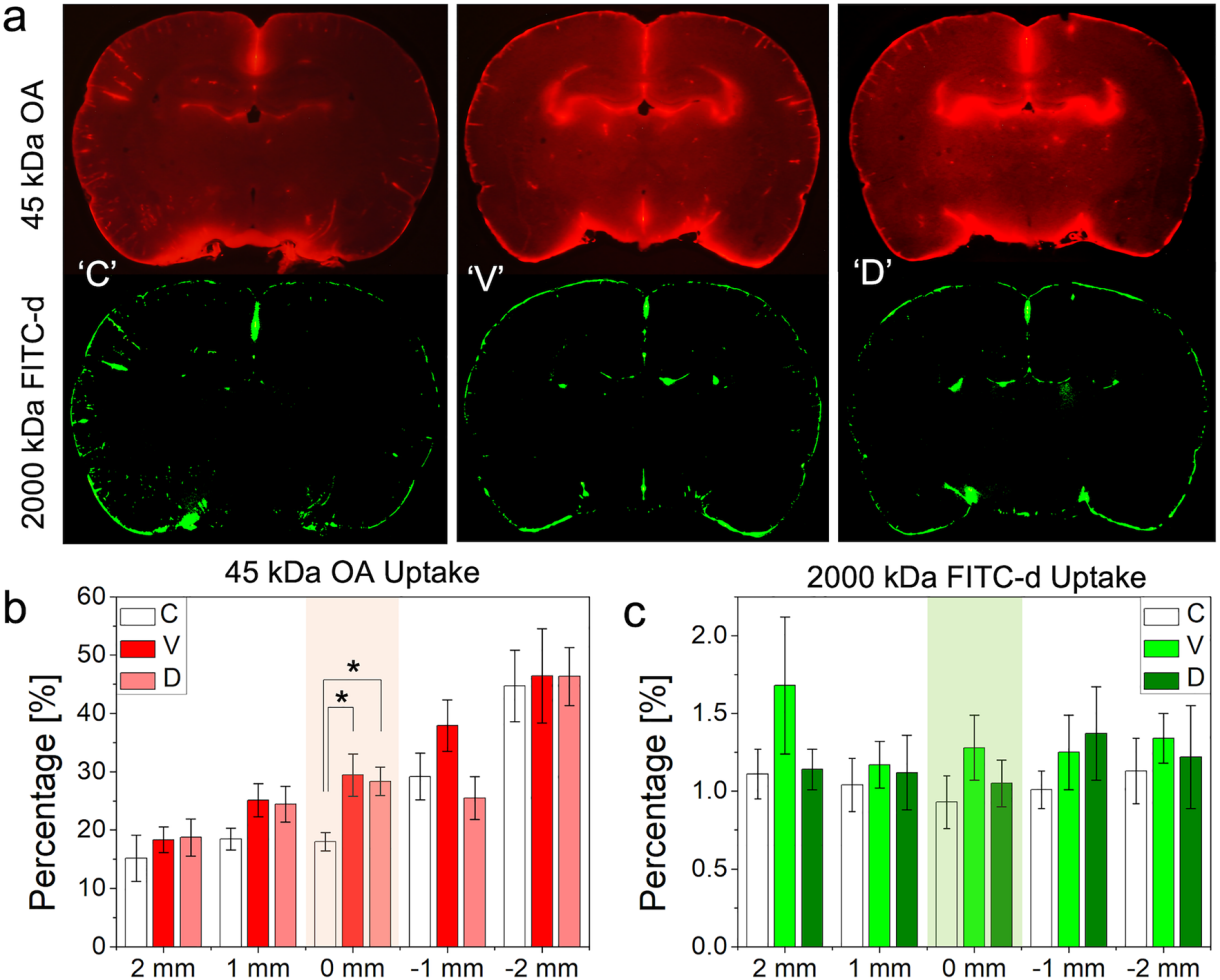
Image analysis of CSF tracer distribution across the experimental condition. (**a**) An exemplar image of OA (in red) and FITC-d (in green) at the coronal planes of sonication across no FUS (‘C’) and FUS conditions (‘V’ and ‘D’). (**b**) % uptake of OA and (**c**) FITC-d (*n* = 6 in each group) across the rostral ( +) and caudal (− ) planes surrounding the sonication (shown in mm;—rostral, + caudal). Colored sections indicate the location of the acoustic focus. *: *P* < 0.05; error bars: standard error.

Fluorescence imaging showed that FUS increased the uptake of OA at the sonicated plane, especially around the ventricles (an example shown in Fig. 4a). Quantitative image analysis across the rostral-caudal sections with respect to the sonicated plane showed that the OA distributed much more extensively in the sonicated plane from FUS conditions (both the ‘V’ and ‘D’ conditions) compared to no sonication (post-hoc Tukey HSD, P < 0.05 following ANOVA; F(2,15) = 5.42, Fig. 4b). The direction of the sonication did not affect the degrees of enhancement (post-hoc Tukey HSD following ANOVA, P = 0.96). The spatial distribution of high M_W_ FITC-d in the brain, on the other hand, was more restricted than that of OA (only ~ 1% uptake) and remained unaffected by the sonication (Fig. 4c; ANOVA, across all sections, F(2,15) < 1.3 and P > 0.32).

### Numerical simulation of thermal analysis

Although low-intensity (I_SPTA_ of 0.5 W/cm^2^) ultrasound was used, we conducted a numerical simulation to model the propagation of acoustic waves in the cranial cavity to evaluate the presence of tissue heating. The simulation was performed using a two-step process: (1) simulation of acoustic propagation and (2) subsequent thermal estimation at the brain tissue as well as at the skull tissue interfaces.

**Figure 5:**
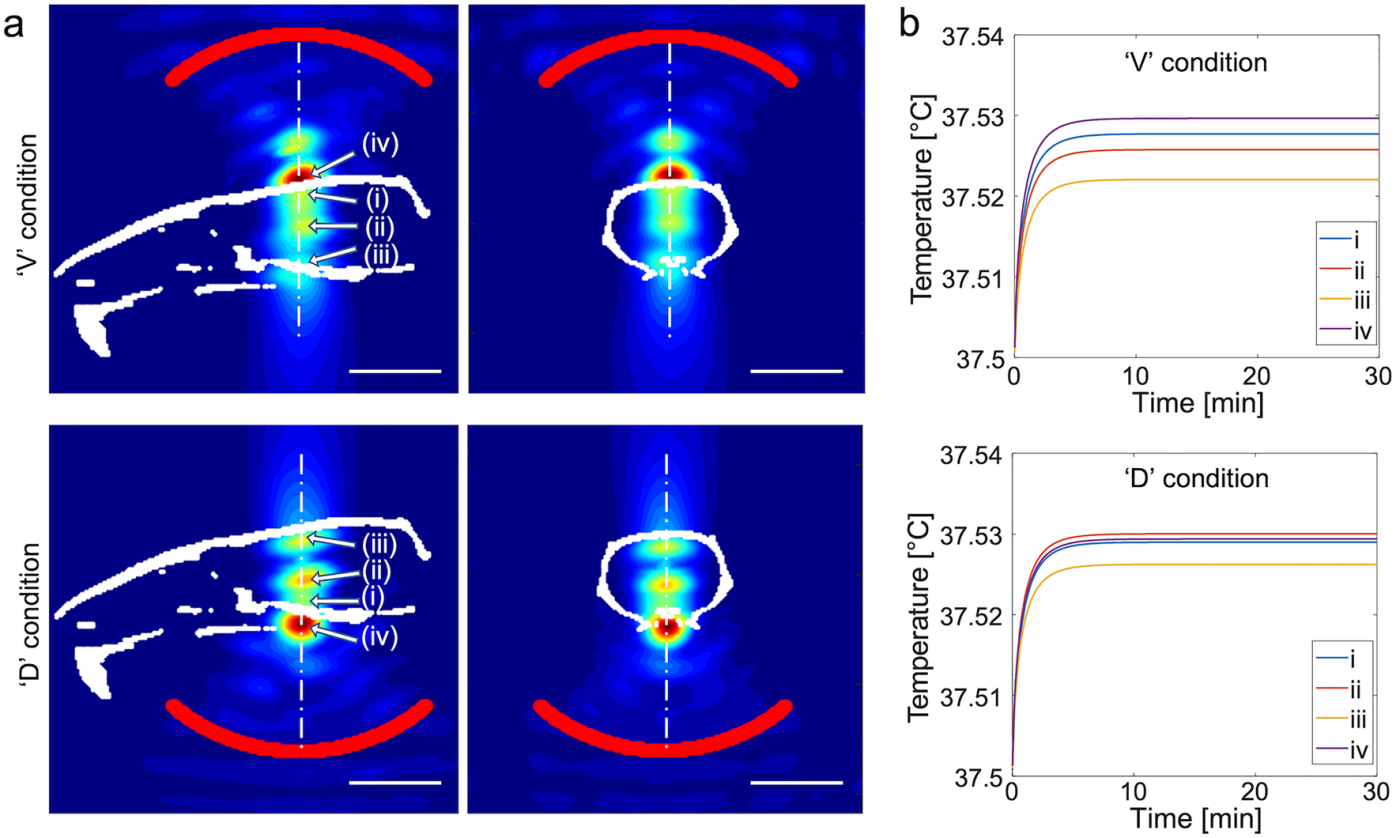
Numerical simulation of the acoustic propagation and thermal rise from different tissue locations. (**a**) Pseudo-colored relative acoustic intensity normalized with respect to the maximum value obtained from the numerical simulation of acoustic intensity along the plane of acoustic focus (obtained from one rat skull, outlined in a white cranial profile) from the ‘V’ and ‘D’ conditions. The profile of the transducer is marked by the red line. Bar = 10 mm. (**b**) Numerical estimation of tissue temperature at (i) the geometric acoustic focus, (ii) local maximum near the center of the brain, (iii) brain-skull interface opposite to the sonication path, as well as (iv) the skull surface facing the incident sonication.

The simulated spatial distributions of the acoustic intensity obtained from a rodent skull are shown at the mid-sagittal/coronal sections along the sonication path for ventral and dorsal FUS conditions (Fig. 5a). In addition to the geometric focal areas (indicated by (i)), the clear presence of reverberation inside the cranial cavity was detected near the midpoint between the skull base and cap (*i.e.*, nearby the ventricles, indicated by (ii)). Another local maximum was seen at the brain tissue interfacing the bone opposite to sonication (iii). The highest intensity occurred at the skull surface facing the incident FUS waves (noted as ‘iv’). This pattern was reproduced in other skull samples, and the averaged I_SPPA_ and peak-to-peak pressure amplitude were tabulated from different locations (‘i’–‘iv’, Table 1). The acoustic reverberation yielded an in situ I_SPPA_ greater than 5 W/cm^2^ at some of these local maxima (*e.g.*, nearby the ventricles and the skull surface facing the incident acoustic waves). Despite reverberations, thermal modeling indicated that the temperature at these areas exhibited only a slight elevation of < 0.03 °C, reaching a steady-state maximum within 10 min of sonication (Fig. 5b).

**Table 1:** Percent intensity transmission (with respect to the free-water maximum of 5 W/cm^2^ I_SPPA_), in situ I_SPPA_ (in W/cm^2^), and peak-to-peak pressure (P, in kPa), numerically estimated at four different locations—(i)–(iv) from Fig. 5. Average ± standard deviation (*n* = 3).

Location	‘V’ condition		‘D’ condition	
	I_SPPA_ (W/cm^2^)	P (kPa)	I_SPPA_ (W/cm^2^)	P (kPa)
i	4.5 ± 0.4	726.7 ± 29.0	4.4 ± 0.1	718.3 ± 8.0
ii	5.1 ± 0.6	775.3 ± 45.8	6.5 ± 0.4	873.7 ± 27.1
iii	3.9 ± 0.7	674.7 ± 62.6	5.4 ± 0.3	801.7 ± 24.6
iv	7.1 ± 0.5	913.3 ± 32.3	7.6 ± 1.6	947.0 ± 103.2

### Histological analysis and evaluation of BBB disruption

**Figure 6:**
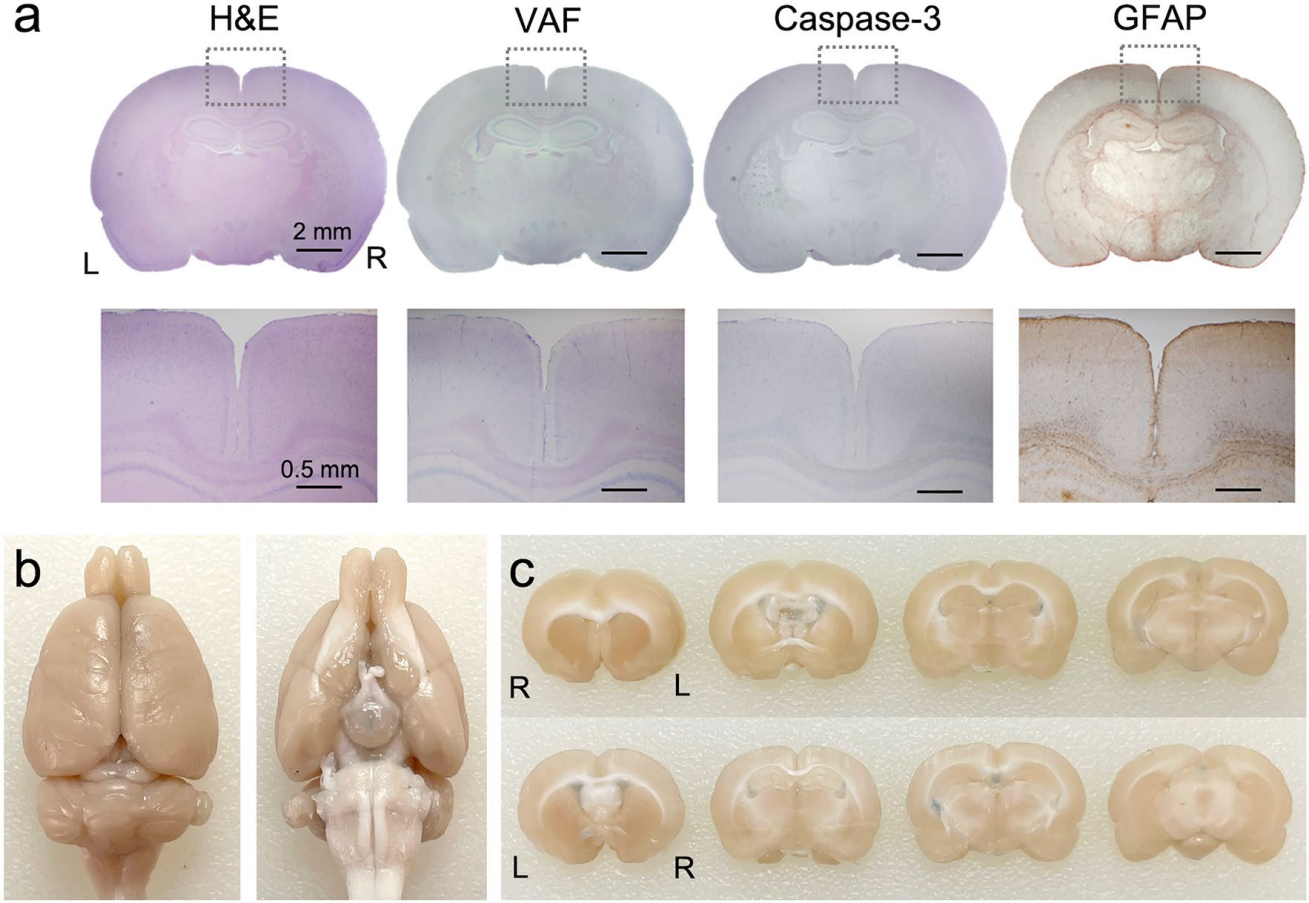
Exemplary histology analysis results of brain tissues and evaluation of blood–brain barrier disruption. (**a**) Top row: H&E, VAF-toluidine blue, caspase-3 and GFAP (from left to right column) stained microscopic images of sonicated brain slices. bar = 2 mm, Bottom row: magnified images from the defined region-of-interest (dotted rectangles). bar = 0.5 mm (**b**) Tail vein injection of trypan blue immediately after FUS sonication revealed no signs of blood–brain barrier disruption in a gross brain specimen and (**c**) 2 mm-thick coronal brain sections shown in rostral and caudal sides. L = left; R = right.

Six male SD rats (weighing 275.5 ± 8.2 g) that did not receive CSF tracer injections were divided into three groups. Two in each group underwent sonication in either ventral or dorsal direction and were subjected to different post-FUS survival duration before sacrifice (immediate, *n* = 2; 2 weeks, *n* = 2; 4 weeks, *n* = 2). The animals’ behavior was monitored during the survival period (30 min observation, 3 days/week). All animals showed normal behavior after the sonication sessions throughout their survival period. The histological analysis of the sonicated brain regions did not reveal any signs of tissue damage in any animal (exemplary data shown in Fig. 6a) in terms of global tissue integrity/hemorrhaging (from H&E), ischemic (VAF-toluidine blue) and apoptotic damage (caspase-3), including glial infiltrations (GFAP). Visual inspection of gross brain anatomy (ventral and caudal view) and coronal brain sections from rats that received an intravenous injection of trypan blue immediately after sonication (*n* = 2) revealed no visible signs of dye extravasation, suggesting the absence of BBB disruption (Fig. 6b).

## Discussion

### Dye infiltration test

Previous studies have shown the utility of the acoustic streaming effect in enhancing drug delivery (*e.g.*, anti-tumor agents) to the brain (referred to as convection-enhanced delivery)^47,48^. Recently, Aryal and colleagues demonstrated that application of diagnostic-intensity FUS operating at 650 kHz in a scanning mode (*i.e.*, continuous sonication along the rectangular trajectory that loops inside of the brain in every 24 s) enhanced the brain parenchymal delivery of intrathecally-injected optical tracers (1 kDa) and panitumumab (150 kDa) in rats^49^. While these studies have provided promising perspectives of using acoustic pressure waves to facilitate CSF solute movement, the acoustic streaming effect involves complicated non-linear fluid dynamics for the transport of small molecules/particles through porous media^24^, especially in the pulsed mode of operation^25,50^. Thus, the effects of pulsing parameters on enhancing TbO dyes to the non-homogenous porous media (melamine foam) were first evaluated, then used to guide the rodent experiment.

Image analysis revealed that the use of 100 ms PD and a 10% DC given at I_SPPA_ of 5 W/cm^2^ yielded the greatest dye infiltration diameter and depth across the tested parameters (Fig. 2e,f). The infiltration was reduced when a PD longer than 100 ms was used, including the continuous wave condition. Lower I_SPPA_ values (≤ 2 W/cm^2^) used under these conditions (to maintain the I_SPTA_ at 0.5 W/cm^2^) suggests that the degree of dye movement was enhanced at a higher I_SPPA_ among the tested parameters. When a fixed PD of 100 ms was applied in different DCs (10–50% with 10% increment by adjusting the PRF), the use of low DCs (10% and 20% given at 5 and 2.5 W/cm^2^ I_SPPA_, respectively) resulted in a greater level of dye infiltration compared to the use of higher DCs (Fig. 2g,h). These findings suggest that the increasing DC alone, given at a constant I_SPTA_, would not enhance acoustic streaming while the use of a higher I_SPPA_ would favor induction of acoustic streaming. The observation also agrees with previous work by Hoyos and colleagues, which estimated the velocity of fluorescent particles (800 nm in diameter) within a microfluidic channel under exposure to pulsed ultrasound waves^25^. However, excessively high I_SPPA_ values would eventually create inertial cavitation, and should be avoided for biological applications.

TbO dye infiltration into the melamine foam became less evident when sonication was delivered to the agar gel blocks (Fig. 2c,d). This suggests that acoustic streaming is less likely to affect the dye movement in materials with sub-micron porosity whereby diffusion becomes the dominant mode of transport over convective bulk flow. Since the agar gel blocks, having a similar porosity to the brain parenchyma (40–80 nm^43,44^), were sonicated to estimate the effects of acoustic streaming, we conjecture that FUS will not likely to influence solute movement within the dense brain neuropil, in which convective bulk flow is not likely to be a dominant source^43,51,52^.

### Enhanced CSF/interstitial tracer movement by tFUS

We acknowledge that the porosity and complexity of the melamine foams cannot replicate the complicated brain cytoarchitecture. Furthermore, the biodistribution of synthetic TbO dyes in the CSF is poorly understood. These limitations necessitated the in vivo testing in rodents using CSF tracers such as OA and FITC-d. Fluorescent images showing the uptake of OA after a 30 min of tracer injection resembled the OA distribution from previous investigations^14,21,51^. Sonication specifically enhanced the OA uptake at the sonicated section, regardless of the sonication direction (ventral or dorsal), showing almost doubled uptake (~ 30% occupancy, Fig. 4b) compared to the no-FUS condition (which showed only ~ 18%, Fig. 4b). Based on the numerical simulation of acoustic propagation (Fig. 5), we found that an additional acoustic focus was formed in the middle of the sonication path in the brain (nearby the ventricles), with an acoustic intensity (thus pressure level) even slightly higher than the intended I_SPPA_. It is likely to stem from constructive interference of incident pressure waves and reflected from the opposite end of the skull; *i.e*., reverberation. The formation of these local pressure maxima might have enhanced acoustic streaming near the ventricle CSF space, further propelling CSF solutes to the surrounding area, yielding the observed OA distribution pattern.

Although the acoustic focus (5 mm in diameter and 15 mm in length, defined at FW_90%_M) was large enough to sonicate nearly the entire brain section in its path, the slices, ≥ 1 mm rostral or caudal to the sonication, did not show differential effects from the sonication. The numerical simulation also revealed that the acoustic focus formed in a restricted area in the rostral-caudal direction, which also support the observed selective OA uptake distribution pattern in the coronal section. Heart rate, which is known to be associated with the degree of solute transport in the brain^18,21,53^, was indifferent across the conditions, thus its contribution to differential OA transport was unlikely. Taken together, these results suggest that FUS enhanced the movement of the OA in the CSF to the brain that received sonication, with high sensitivity to the spatial intensity profile.

We note that the fixation method employed (paraformaldehyde (PFA) perfusion) is known to greatly reduce the volume of PVS^18^, thus confounding the interpretation of fluorescence imaging. The PFA perfusion fixation, uniformly adopted across the experimental conditions, would not have impacted the interpretation of the results, but may have reduced the observed fluorescence level in the brain tissue. Use of imaging techniques such as in vivo two-photon microscopy^18^ or infrared confocal fluorescence imaging^54^ would be helpful to monitor CSF tracer movement in live animals as well as to probe the contribution from potential expansion of the extracellular and perivascular space that may accompany pulsed application of ultrasound^55^.

The use of low frequency (200 kHz) ultrasound waves in the present study, albeit advantageous for transcranial application, was prone to generating intracranial reverberations due to the longer wavelength compared to the rodent cranial dimension. The use of an animal model having larger cranium, such as sheep or non-human primates would be beneficial to reduce/eliminate reverberation. In fact, the 200 ~ 300 kHz frequency range has been safely used in studies of FUS-mediated functional brain neuromodulation among sheep^40^ and humans^56^ without the effects of reverberation. Alternatively, the use of higher frequency ultrasound can be sought after, especially applicable to assessment among small animals, with added potential merits in creating acoustic streaming effects at higher efficiency^25^. The use of higher frequency (650 kHz) ultrasound, operating at a lower intensity, DC (7.7%), and a shorter sonication duration (10 min) than the present study, has shown to enhance the CSF solute uptake far greater than the one observed herein^49^. Since acoustic streaming is also known to be governed by the wavelength and amplitude shape of acoustic pressure waves^57^, the effects of pulsing scheme, including the effects of amplitude-modulation of acoustic waveforms (other than standard sinusoidal waves), on FUS-mediated CSF solute transport warrant further investigation.

The distribution of high M_w_ (2000 kDa) FITC-d occupied much smaller areas than the uptake of OA (~ 1% uptake per given section of the brain area), which suggests that CSF solute movement is size-dependent. The results matched well with previous investigations showing inferior efficiency in transporting large exogenous macromolecules^14,51,58^ which shared clinical relevance to the accumulation of large aβ plaques and fibrils in AD^59,60^. Unlike 45 kDa OA with its movement enhanced by the sonication, FUS did not affect the movement of high M_w_ (2000 kDa) FITC-d in the present study (Fig. 4c), which suggests the presence of size limit of solutes that can be transported by the FUS. 150 kDa-sized intrathecally-injected panitumumab, much bigger than 45KDa OA, has shown to be efficiently transported to the brain parenchyma through the application of FUS^49^, and calls for further investigation of gauging the effects of FUS on moving various types and size of CSF solutes.

To estimate the solute movement patterns enhanced by the sonication, a numerical approach to model the convective flow associated with the acoustic streaming effects can be considered. However, acoustic streaming, especially in non-homogenous media such as the brain, would involve highly complicated nonlinear fluid dynamics that depend on many unknown acoustic properties in brain tissue^24,25,50^. In addition, complex and inhomogeneous brain cytoarchitecture are largely unknown, including anisotropic porosities and spatial orientations of specific neuropil as well as structures of perivascular CSF space^43^. Furthermore, contribution of the arterial pulsation on the CSF flow patterns^18^ is another important factor to be considered in numerical estimation. Thus, development of numerical simulations describing the effects of acoustic streaming on CSF solute transport within the entire brain volume would meet great challenges and constitutes a subject for future investigation.

### Thermal analysis and safety considerations

Thermal simulations showed that low-intensity sonication used in the present work is unlikely to cause temperature elevation in the brain tissue, despite the formation of intracranial acoustic foci having a slightly higher acoustic intensity. To ensure the absence of thermal confounders, magnetic resonance imaging (MRI)-based thermometry^61,62^ can be adopted to monitor temperature changes caused by sonication in vivo. Nevertheless, comprehensive histological analyses obtained from the animals that received sonication (Fig. 6a), along with the observed normal animal behavior, attest to the safety of the transcranial application of pulsed FUS to the rat brain.

Regarding the mechanical impact on biological tissue, especially associated with undesirable cavitation, the use of a 386 kPa peak rarefactional pressure (P_r_) in the present study did not yield signs of brain tissue damage. Although the cavitation threshold for the brain tissue was not measured from the present study, approximately 580 kPa P_r_ produced cavitation in an agar phantom^63^ (3 s continuous waves at 1 MHz) while ~ 800 kPa P_r_ (continuous 1 MHz sonication) was estimated to generate cavitation in water, as predicted by theoretical models^64^. These P_r_ values are much higher than the one used in the present study, which support our observation that cavitation was unlikely. As the application of longer sonication pulses tends to induce cavitation at a lower P_r_ than a single pulse^64^, further study on the systematic evaluation of cavitation threshold in the brain tissue across the different PDs and frequency range would be helpful in gauging the safety of long pulse ultrasound.

### Potential application to promote waste removal from the brain

We found that non-thermal tFUS sonication facilitated regional CSF movement of 45 kDa-M_w_ OA in the rodent brain without disrupting the BBB (Fig. 6b). This led us to believe that the technique can be used to facilitate waste clearance from the brain, including similar-sized Aβ monomers and oligomers (precursors for plaque formation^46^) that are associated with Alzheimer’s disease (AD). Regional enhancement of CSF solute clearance, especially on Aβ, has been demonstrated in a murine model of early dementia^65^ by actively disrupting the BBB using intravenous ultrasound microbubble with concomitant application of FUS. However, the technique is vulnerable to potential risks for cavitation-related tissue damage (*e.g.*, hemorrhaging^66,67^) as well as modification of local hemodynamic properties^68–70^. If shown to facilitate the solute removal from the brain without disrupting the BBB, the present method may provide elegant and unprecedented means to enhance clearance of waste products from the brain, including Aβ/tau proteins. Further investigation is needed to examine the detailed effects of sonication parameters on promoting the clearance of interstitial tracers, *e.g.*, intracortically injected radio/fluorescent-labeled Aβ oligomers.

As a completely non-invasive technique, we envision that transcranial FUS can be applied to a wide spectrum of neurological conditions associated with aberrant brain lymphatic function and suboptimal clearance of the metabolic waste. For example, compromised local fluid-exchange inside the infarct core due to stroke lead to the accumulation of proinflammatory cytokines, causing brain inflammation and neurodegeneration^71–73^. Furthermore, as the white matter tracts in the brain consist of myelinated nerve bundles that allow for anisotropic fluid movement^74,75^, enhancement of convective CSF motion along the tracts may also be conceived as a future extension of the technique for possible amelioration of the impaired lymphatic clearance implicated with multiple sclerosis^76,77^. It is also plausible to conjecture that facilitated lymphatic function of the brain may compensate for suboptimal clearance of metabolites burden (*e.g.*, lactate and Aβ), associated with sleep deprivation^78–80^.

New findings in the present study are summarized herein. First, a specific pulsing scheme (i.e., 100 ms PD operating at 10% DC) provided superior transport efficiency than continuous waves based on the dye infiltration test. Secondly, the technique can regionally transport CSF solutes (OA) having a much higher M_W_ than most CNS drugs (typically < 1 kDa), which have been transported by existing CED techniques. On the other hand, there was a limitation on the molecule size for our technique to enhance the transport (2000 kDa dextran). The FUS-mediated regional transport of OA, bearing size similarity to the neuroactive Aβ oligomers in AD (sized ~ 8–70 kDa), would become particularly important, for example, for enhancing the transport of Aβ proteins that preferentially accumulate in a region-specific fashion^81^.

## Conclusions

We have demonstrated that localized application of pulsed tFUS safely promoted the movement of intracisternally injected CSF tracers into the rat brain parenchyma. Although the transport of solutes in the CSF plays an instrumental role in their clearance from the brain, further investigation of the effects of tFUS on directly promoting solute clearance from the brain is urgently needed, including examination of the effects of sonication parameters beyond those tested, to position tFUS as new neurotherapeutic platform.

## Methods

### FUS transducer

A FUS transducer (GS200-D25-P38, Ultran Group, State College, PA) was actuated by a sinusoidal electrical waveform amplified by a linear power amplifier (240L, Electronics and Innovations, Rochester, NY). A function generator (33500B, Keysight, Santa Rosa, CA) was used to produce the initial waveform. The spatial distribution of the acoustic intensity profile was characterized in degassed water tank using a method described elsewhere in detail^62,82^. The FUS transducer was immersed in degassed water (< 2 ppm oxygen level; measured by colorimetric K-7512 ChemMets kit, Chemetrics, Midland, VA), and the longitudinal pressure profile along the sonication axis was mapped using a needle hydrophone (HNC-200, Onda Corp, Sunnyvale, CA) mounted to a robotic, 3-axis linear stage (Bi-Slides, Velmex Inc., Bloomfield, NY) with a 1 mm step covering a 5 cm × 3 cm rectangular area (5 mm away from the exit plane of the transducer). The focus was defined at the location having the maximum pressure, and the pressure profile perpendicular to the acoustic focus was also measured with a 1 mm step covering 3 cm × 3 cm. The pressure at the focus was calibrated with respect the input voltage using a calibrated hydrophone (HNR-500, Onda Corp, Sunnyvale, CA).

### Melamine foam and agar hydrogel blocks for dye infiltration tests

Melamine foams (IEXL21, Sponge outlet, Kenmore, NY), having pore diameter range of ~ 10–100 μm, were cut 50 mm × 45 mm × 35 mm (width × height × thickness). Agarose power (A4018, low gelling temperature, Millipore Sigma) was dissolved in PBS at concentration of 2.5% w/w in 50 °C. The solution was poured into a 30 × 30 × 10 mm (width × height × thickness) mold and gelled^83^. The melamine foam and the agar block were inserted into a chamber that was 3D-printed (Form3, Formlabs, Somerville, MA). The chamber contained guide slots to position the acoustic focus at 1 mm in front of the surface of the block (by introducing a 12 mm gap between transducer exit plane and the surface; Fig. 1c,d).

### Rodent FUS platform

The animal was positioned over an in-house sonication setup that consisted of a vertical stage that moved the stereotactic platform and a two-axis stage which controlled the transducer position in horizontal directions (each stage had 15 µm spatial precision, NLS 4, Newmark Systems, CA). The bregma location for referencing the sonication location was estimated 9 mm rostral to the interaural line.

### Animal procedures and intracisternal injection of CSF tracers

The present study was carried out and reported in accordance with the ARRIVE guidelines (https://arriveguidelines.org/). All animal procedures were conducted in compliance with the Institutional Animal Care and Use Committee (IACUC) regulations and standards, set forth by the Brigham and Women’s Hospital. Sprague–Dawley (SD) rats (all male) were socially housed (two rats/cage) under a 12 h/12 h light/dark cycle (lights on at 7 AM, off at 7 PM). The rats were allowed to access food and water ad libitum.

As the CSF tracers, 45 kDa-M_W_ Texas Red Ovalbumin (OA, 023,021, Thermo Fisher) and 2000 kDa-M_W_ fluorescein isothiocyanate (FITC)-dextran (FITC-d, FD2000S, Millipore Sigma) were constituted at a 0.5 wt% concentration in artificial CSF (aCSF; Tocris Bioscience, Bristol, UK). The prepared CSF tracer solution were intracisternally injected based on an established surgical protocol^45^. Rats (*n* = 6 in each group, total 3 groups) were anesthetized with an intraperitoneal injection of a mixture of 40–80 mg/kg ketamine and 10 mg/kg xylazine, then placed on a stereotactic frame (ASI Instruments, Warren, MI). Respiratory rate was measured manually and additional doses (~ half dose each) of anesthesia were administered as needed. After adequate anesthesia was achieved, the animal’s head was shaved using a clipper and depilation lotion. A temperature-controlled water-circulating pad (T-pump, Gaymar, Orchard Park, NY) was placed under the animal to maintain the animal’s body temperature.

Saline and/or cotton swabs were used to clean and dry the dura covering the cisterna magna (CM). A 30G cannula, filled with aCSF, was inserted into the center of the subarachnoid CM space at a depth of 1–2 mm, perpendicular to the covering dura. The needle insertion site was sealed with a few drops of cyanoacrylate glue (with application of accelerant). The CSF tracers were injected at a rate of 2 µL/min for 10 min using a syringe pump (Legato 100, KD Scientific, Holliston, MA), whereby the injection rate is known not to elevate the intracranial pressure^21^.

During the time to allow CSF tracers to distribute across the brain (with and without application of FUS), Respiratory and heart rates were measured (measured by Surgivet Hand-held pulse oximeter V1030, Smiths Medical, Dublin OH) right before and after the FUS, as heart rate is a critical factor known to affect CSF transport.

### Fluorescent image analysis

Immediately after a FUS sonication session, the animal’s brain was harvested upon transcardial perfusion of 4% paraformaldehyde (PFA) in PBS (BM-155, Boston Bioproducts, Ashland, MA) and additional PFA submerge fixation (~ 24 h). The extracted brain underwent vibratome sectioning in 200 µm-thick slices (PELCO easiSlicer, Ted Pella, Redding, CA) along the rostral-caudal direction encompassing the acoustic focus. The sectioned slices were imaged with a widefield fluorescent microscope (TS100, Nikon, Japan) using an ultrawide field (23.4 × 15.6 mm) CMOS Sensor (4592 × 3056 pixel resolution, NEX-5, Sony, Japan). The sectional images from 2 mm rostral to 2 mm caudal directions from the sonication location with 1 mm gap in-between (thus, a total of 5 images) were normalized to the intensity scale ranging from 0 to 255 (8-bit image) and were processed with ImageJ software (version 1.53i, https://imagej.nih.gov/ij/)^84^. The brain area was first segmented, guided by the background fluorescence and corresponding bright-field image. The areas showing tracer uptake were segmented above 170% of the minimum background fluorescence value. Then, the percent area of tracer uptake with respect to the brain was derived from each animal for OA and FITC-d images and underwent one-way ANOVA followed by Tukey HSD *post-hoc* test for multiple comparisons.

### Numerical simulation acoustic propagation and thermal analysis

For the acoustic simulation, the three-dimensional (3D) skull geometry was obtained from ex vivo rat skulls (*n* = 3; without the mandibular bones) using computed tomography (CT, SkyScan micro-CT, Source 60 kV, Bruker, Billerica, MA, USA) in isotropic voxels of 17 × 17 × 17 μm^3^. Hounsfield unit (HU) values in the images were calibrated with respect to a water tube located within the same image volume. The acquired images were subsampled to make isotropic voxels of 0.25 μm on each side, yielding a ratio of 30 pixels per wavelength in water (λ =  ~ 7.5 mm at 200 kHz) to attain sufficient spatial resolution for the simulation^85^. The acoustic source was modeled based on our previous method^82^, factoring in the transducer geometry (transducer diameter of 28 mm and curvature radius of 22 mm), and positioned in the location used in the experiment. A simulation volume encompassing both the transducer and skull (183 × 275 × 266 voxels, 45.75 × 68.75 × 66.5 mm^3^) was used in the finite-difference time domain (FDTD)-based analysis with a time resolution of 0.05 μs. The acoustic properties of the skull, *i.e.*, speed of sound, density, and attenuation, were used as described in our previous work^86^ based on the HU values of CT images^87^.

The resulting 3D distribution of acoustic intensity was used to estimate the temperature change at the skull and brain tissue by solving the bio-heat transfer equation^55,87^^88^ based on an FDTD scheme with discretized spatial resolution of 0.25 mm and a time resolution of 0.5 ms. The sonication parameters from the in vivo study (fundamental frequency of 200 kHz, I_SPPA_ of 5 W/cm^2^, 100 ms PD, 1 Hz PRF [*i.e.*, 10% DC] and 30 min sonication duration) were used in the estimation assuming homogenous intracranial brain tissue and an initial tissue temperature of 37.5 °C. Thermal properties of the brain (specific heat of 3600 J/kg/K, thermal conductivity of 0.528 W/K/m), skull (specific heat of 1300 J/kg/K, thermal conductivity of 0.4 W/K/m) and blood perfusion (perfusion rate of 8.24 kg/m^3^/s, density of 1030 kg/m^3^, specific heat of 3620 J/kg/K) were assigned to the simulation^89^. Contributions from the pulsation of CSF and thermoregulatory responses in heat transfer/removal^90^ were not modeled to conservatively overestimate the temperature rise in the brain tissue.

### Histological analysis and evaluation of BBB disruption

We transcardially perfused the rats that were not injected with the CSF tracer (4% formaldehyde) and extracted brain after additional 24 h immerse fixation. The sonicated brain area was sectioned and stained with hematoxylin and eosin (H&E) to detect necrosis or hemorrhaging, vanadium acid fuchsin (VAF)-toluidine blue to detect the presence of ischemic neurons, glial fibrillary acidic protein (GFAP) immunohistochemistry (IHC) to detect glial infiltration, and caspase-3 for visualization of any apoptotic activity^91^. The integrity of the BBB was separately assessed (from 2 rats) using intravenous (via tail-vein injection) trypan blue (in saline, 0.1 g/kg, 872.9 Da) perfusion^85^ for 30 min right after tFUS (*n* = 2, each receiving sonication from dorsal or ventral direction). Transcardial perfusion fixation was performed using the same fixation protocol, and the brain was subsequently harvested and imaged using a dissection microscope.

## Data Availability

The datasets used and/or analyzed during the current study available from the corresponding author on reasonable request.
